# Function of a p24 Heterodimer in Morphogenesis and Protein Transport in *Penicillium oxalicum*

**DOI:** 10.1038/srep11875

**Published:** 2015-07-07

**Authors:** Fangzhong Wang, Kuimei Liu, Lijuan Han, Baojie Jiang, Mingyu Wang, Xu Fang

**Affiliations:** 1 State Key Laboratory of Microbial Technology, School of Life Science, Shandong University, Jinan, Shandong, China

## Abstract

The lignocellulose degradation capacity of filamentous fungi has been widely studied because of their cellulase hypersecretion. The p24 proteins in eukaryotes serve important functions in this secretory pathway. However, little is known about the functions of the p24 proteins in filamentous fungi. In this study, four p24 proteins were identified in *Penicillium oxalicum.* Six p24 double-deletion strains were constructed, and further studies were carried out with the *ΔerpΔpδ* strain. The experimental results suggested that Erp and Pδ form a p24 heterodimer *in vivo.* This p24 heterodimer participates in important morphogenetic events, including sporulation, hyphal growth, and lateral branching. The results suggested that the p24 heterodimer mediates protein transport, particularly that of cellobiohydrolase. Analysis of the intracellular proteome revealed that the *ΔerpΔpδ* double mutant is under secretion stress due to attempts to remove proteins that are jammed in the endomembrane system. These results suggest that the p24 heterodimer participates in morphogenesis and protein transport. Compared with *P. oxalicum Δerp*, a greater number of cellular physiological pathways were impaired in *ΔerpΔpδ*. This finding may provide new insights into the secretory pathways of filamentous fungi.

The depletion of fossil fuels is increasing the need to find alternative energy sources. Biofuels are promising alternatives to fossil fuels. The production of biofuels is friendly to the environment and primarily requires renewable resources^1^. These resources include abundant lignocellulose. However, the high cost of lignocellulose-degrading enzymes hinders the industrial biorefinery of lignocellulose^2^,^3^. Many studies have focused on the production of lignocellulose-degrading enzymes derived from filamentous fungi, which exhibit high cellulolytic enzyme activity. Previous studies in this field concentrated on the following aspects: 1) cellulase synthesis and transcriptional regulation mechanisms^4^,^5^,^6^, 2) biochemical properties of lignocellulolytic enzymes^7^,^8^, and 3) screening and optimisation of fermentation conditions for cellulase-hyperproducing strains^9^,^10^. Filamentous fungi demonstrate outstanding extracellular protein production capabilities^3^,^4^,^6^,^9^,^10^. However, few studies have been carried out to examine the secretory pathways in filamentous fungi.

Recent studies have attempted to elucidate the secretion mechanisms of filamentous fungi. For example, exocytosis was found to occur not only at the hyphal tip of fungi but also at the septa or subapical regions^11^,^12^. Cargo transport in filamentous fungi from the endoplasmic reticulum (ER) to the plasma membrane occurs through a Golgi-like apparatus with the aid of F-actin and microtubules. However, this mechanism is gradually being overshadowed by the discovery of unconventional secretion mechanisms. Giraldo *et al.* (2013) demonstrated that some cargoes that enter the ER are directly released into the extracellular space without passing through the Golgi-like apparatus^13^. Stock *et al.* (2012) demonstrated that the secretion of endochitinase in *Ustilago maydis* is independent of the ER and the Golgi-like apparatus^14^. Many proteins located in the secretory pathway of filamentous fungi have been characterised. Examples of these proteins are chaperones (BiP and PDI) that aid in the correct folding of proteins and SNARE families that promote the fusion of vesicles with targeted membranes^15^,^16^. However, only a few studies have analysed the p24 family in filamentous fungi.

The family members of p24 are 24-kDa type-I transmembrane proteins that may be further divided into four p24 subfamilies (α, β, γ, and δ), the composition of which varies among species^17^. The localisation of p24 proteins varies between different subfamilies or even within the same subfamily^18^. The functional divergence of p24 proteins among different subfamilies was discovered in *Xenopus laevis*^19^. The deletion of certain p24 proteins in yeast delays the anterograde transport of invertase and the glycosylphosphatidylinositol-anchored protein Gaslp but does not affect other cargo molecules (α-factor, acid phosphatase)^20^,^21^. Therefore, the role of p24 proteins in cargo recruitment maybe result in the selection of specific cargoes to be transported into certain vesicles. In yeast, the p24 proteins select correctly remodelled GPI-anchored proteins for transport into the COPII vesicle and simultaneously retrieve and transport escaped, unremodelled GPI-anchored proteins back to ER in the COPI vesicle^22^. Thus, it has been suggested that the p24 proteins mediate quality-control mechanisms in GPI-anchored protein transport. Several reports indicate that the p24 proteins may be involved in the biogenesis of COPI and COPII vesicles^17^. The p24 proteins usually contain similar architectural structures, such as the N-terminal GOLD domain, which has been predicted to mediate protein-protein interactions^23^, the coiled-coil region, presumably involved in p24 oligomer assembly^24^, the transmembrane-spanning domain, and a cytoplasmic tail involved in COPI and COPII binding^25^,^26^.

*Penicillium oxalicum* 114-2 was originally isolated from decayed straw-covered soil in China in 1979^3^. *P. oxalicum* 114-2 has many advantages in terms of lignocellulose degradation over *Trichoderma reesei*, which is used as the principal commercial producer of cellulase^3^. The whole-genome sequencing of *P. oxalicum* was completed in 2014^6^. However, little is known about the genes predicted in the secretory pathways.

The roles of Erp were identified in *T. reesei* and *P. oxalicum*^27^. However, other p24 proteins have yet to be studied in *P. oxalicum*. Jenne *et al.* (2002) demonstrated that the p24 family members are usually present as dimers or monomers, depending on the p24 protein and its subcellular location^28^. Four p24 proteins were identified in *P. oxalicum*. Of these, further studies were carried out with Erp and Pδ in this work that suggested the formation of a p24 heterodimer *in vivo*. The physiological functions of the p24 heterodimer were investigated. A comparison between *P. oxalicum Δerp* and *ΔerpΔpδ* was conducted. This study is the first report of the physiological roles of a p24 heterodimer in filamentous fungi.

## Results

### p24 Homologs in *P. oxalicum* 114-2

**Figure 1:**
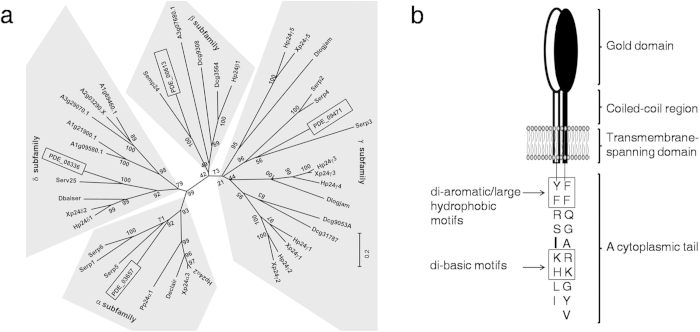
Erp and Pδ Homologs in *P. oxalicum* 114-2. Panel **a**, phylogenetic tree of certain p24 proteins from eukaryotic species. Black rectangles represent PDE_09471, PDE_08336, PDE_00613 and PDE_03657. H, *Homo sapiens*; D, *Drosophila melanogaster*; S, *Saccharomyces cerevisiae*; X, *Xenopus tropicalis*; P, *Pan troglodytes*; A, *Arabidopsis thaliana.* Protein accession numbers are listed in Supplementary Information S1 online. Panel **b**, structural diagram of the p24 heterodimer, which is composed of Erp and Pδ. White represents Pδ, and black represents Erp. Each protein domain is marked with a bracket. The di-aromatic/large hydrophobic and di-basic motifs are highlighted with rectangles.

A BlastP search was performed against the *P. oxalicum* 114-2 genome with *Saccharomyces cerevisiae* Emp24, Erv25, Erp3, and Erp5 as queries, from which PDE_00613 (5e-46 E-value, 46% identity), PDE_08336 (3e-55 E-value, 50% identity), PDE_09471 (4e-26 E-value, 31% identity), and PDE_03657 (2e-41 E-value, 38% identity) were identified as the best hits, respectively. A BlastP search was also performed against the *S. cerevisiae* genome with PDE_00613, PDE_08336, PDE_09471, and PDE_03657 as queries, during which the best hits obtained were Emp24 (3e-46 E-value, 46% identity), Erv25 (5e-55 E-value, 50% identity), Erp3 (7e-26 E-value, 50% identity), and Erp5 (3e-41 E-value, 38% identity), respectively. The sequence alignment of these proteins is shown online in Supplementary Information Fig. S1. Phylogenetic analysis of the four proteins in comparison with other p24 homologs suggested that PDE_03657, PDE_00613, PDE_08336, and PDE_09471 belong to the α, β, δ and γ subfamilies, respectively (Fig. 1a). Six p24 protein double deletion strains were constructed and confirmed by PCR. Their intracellular proteins were analysed using 15% SDS-PAGE. The protein bands of the various p24 deletion strains appeared to be different. The strain that lacked PDE_09471 and PDE_08336 exhibited the most significant changes in comparison to the parent strain (see Supplementary Information Fig. S2 online). As a result, PDE_09471 and PDE_08336 were selected for further investigation. PDE_09471 has been described in detail^27^ and has been termed Erp. The PDE_08336 was termed Pδ to be consistent with the nomenclature of the p24 family. The ORF of *pδ* is 892 bp long and is interrupted by two introns^6^. As it is known that members of the p24 family usually share similar topologies^29^, the structure diagrams of Erp and Pδ (Fig. 1b) were constructed by comparing their sequences with those of other p24 members. The conservative motifs predicted to be involved in COPI and COPII binding are marked with rectangles in Fig. 1b^17^,^26^,^30^.

### Erp-Pδ interaction studies *in vivo*

**Figure 2:**
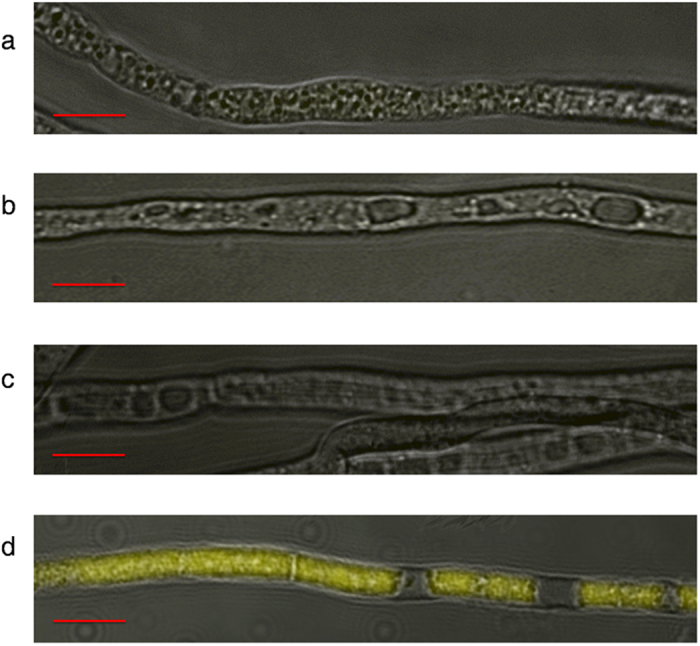
Visualisation of the Erp-Pδ Interaction Through the BiFC Assay. Strains carrying **a**) *eyfpN + eyfpC*, **b**) *eyfpN* + *pδ*-*eyfpC*, **c**) *erp*-*eyfpN* + *eyfpC*, and **d**) *erp*-*eyfpN* + *pδ*-*eyfpC*. Scale bars: 10 μm.

Bimolecular fluorescence complementary analysis (BiFC) was performed to test the Erp-Pδ interaction *in vivo*. There were three negative controls (the strains expressing *eyfpN + eyfpC*, *eyfpN* + *pδ*-*eyfpC, erp*-*eyfpN* + *eyfpC*) in this experiment. None of them fluoresced (Fig. 2a–c). These results indicate the absence of autofluorescence or nonspecific background in the BiFC assay. However, the hyphae of the strains expressing *erp*-*eyfpN* and *pδ*-*eyfpC* exhibited yellow fluorescence (Fig. 2d). The yellow fluorescence in Fig. 2d suggests that Erp and Pδ are spatially close and interact with each other, forming a heterodimer in *P. oxalicum*.

### Phenotypic and Quantitative Conidiation Assays

**Figure 3:**
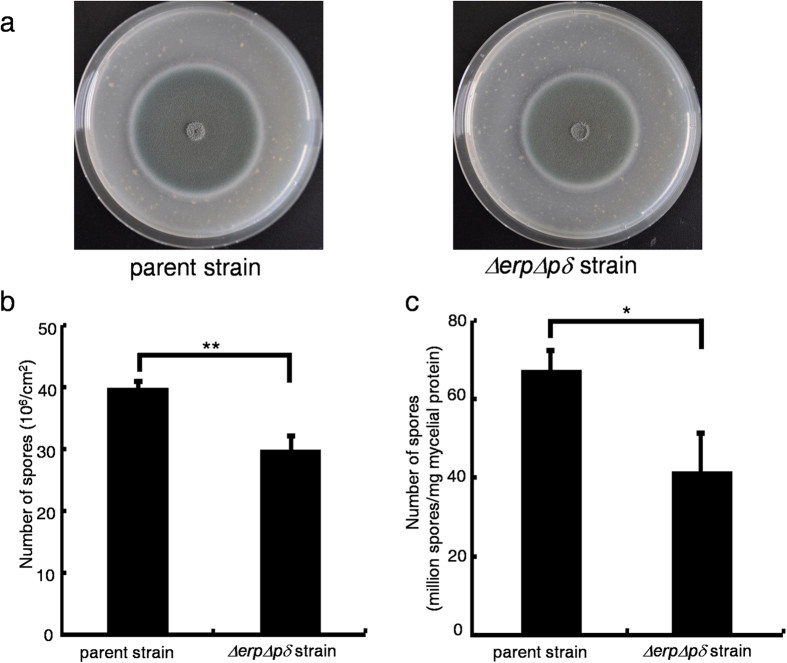
Comparison of the Sporulation Efficiencies of the Parent and *ΔerpΔpδ* Strains. *P < 0.05, **P < 0.01. Panel **a**, phenotypic analysis of the parent and *ΔerpΔpδ* strains on wheat bran plates. Panel **b**, the number of spores produced per square centimetre on wheat bran plates. Panel **c**, the number of spores produced per milligram of mycelial protein in submerged cultivation. Error bars indicate the standard deviation calculated from three biological experiments.

The roles of Erp in *T. reesei* and *P. oxalicum* were investigated. Sporulation on wheat bran plates was drastically reduced for *T. reesei Δerp* but not *P. oxalicum Δerp* (spore production was quantified by counting the number of spores per square centimetre using a haemocytometer)^27^. Therefore, approximately 1 × 10^7^ spores were inoculated onto wheat bran plates at 30 °C for 3 d to detect whether the sporulation pathway was impaired in the *ΔerpΔpδ* strain (see Supplementary Information Fig. S3 online for Southern blotting analysis of *pδ*). The number of spores was significantly reduced in the *ΔerpΔpδ* strain (P = 0.0054, n = 18) (Fig. 3b). To further confirm this result, comparison of the sporulation levels of the parent and *ΔerpΔpδ* strains during submerged cultivation was performed. The number of spores was significantly decreased for the *ΔerpΔpδ* strain (P = 0.017, n = 5) (Fig. 3c). Therefore, this finding suggests that the *ΔerpΔpδ* strain is likely to be defective in sporulation.

The colony diameter was slightly reduced for *P. oxalicum Δerp*, while it was considerably decreased for *ΔerpΔpδ* [parent strain: 21 ± 0.5 mm (mean ± s.d., n = 3), *ΔerpΔpδ* strain: 15 ± 0.4 mm (mean ± s.d., n = 3)] (Fig. 3a). These results indicate that the p24 heterodimer participates in conidiospore formation and hyphal growth.

### Comparison of Lateral Branching Development Patterns Between the Parent and *ΔerpΔpδ* Strains

**Figure 4:**
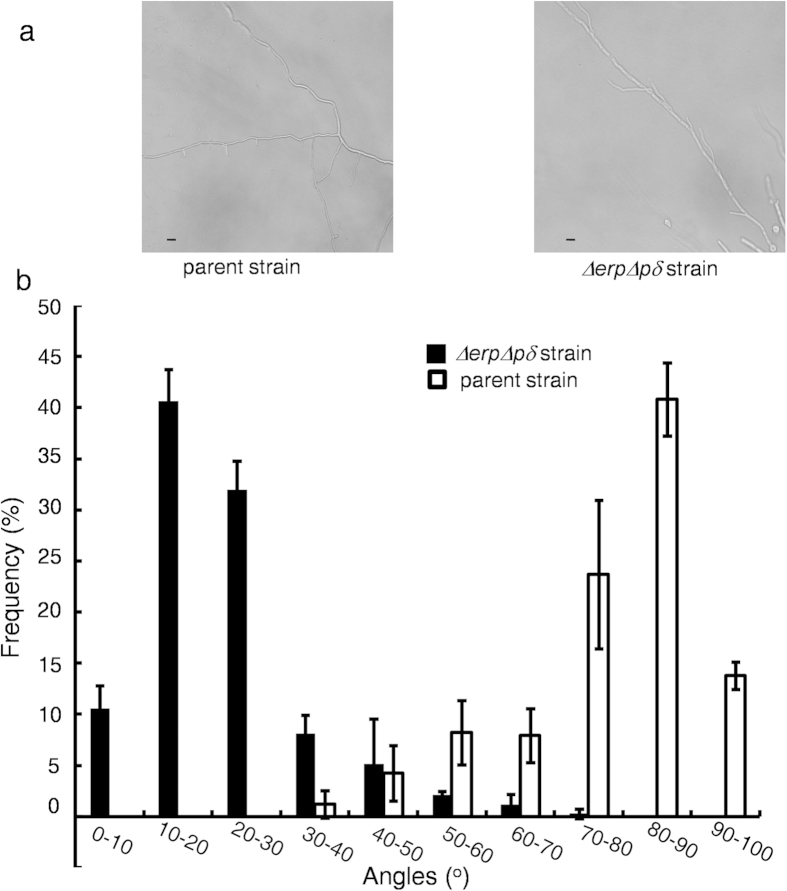
Comparison of Lateral Branching Development Patterns Between the Parent and *ΔerpΔpδ* Strains. Panel **a**, microscopic observation of hyphae from the parent and *ΔerpΔpδ* strains. Scale bar: 10 μm. Panel **b**, statistical comparison of angle distribution between main hyphae and lateral branches in the parent and *ΔerpΔpδ* strains. White represents the parent strain, and black represents the *ΔerpΔpδ* strain. Error bars indicate the standard deviation calculated from three biological experiments.

An increased branch frequency with dichotomous tips was observed for *T. reesei Δerp* but not *P. oxalicum Δerp* when the strains were cultivated on agar plates containing 2% glucose. This indicates that the branching mechanism was disrupted in *T. reesei Δerp*. To test whether branching was affected in the *ΔerpΔpδ* strain, approximately 1 × 10^3^ spores were inoculated on glass slides covered by glucose-containing agar media. There are two types of branching: tip and lateral branching. Compared with the parent strain, lateral branching but not tip branching was drastically altered in the *ΔerpΔpδ* strain (Fig. 4a). The angles between the main hyphae and lateral branches in the parent and *ΔerpΔpδ* strains were statistically analysed (Fig. 4b). The main frequency distribution of the branching angles ranged from 70° to 90° in the parent strain and 10° to 30° in the *ΔerpΔpδ* strain. The branching angle in the parent strain was 77.78° ± 13.68° (mean ± s.d., n = 300), while in the *ΔerpΔpδ* strain it was 21.82° ± 11.89° (mean ± s.d., n = 300). These results suggest that the p24 heterodimer participates in lateral branching.

### Assessment of the Protein Secretion Capabilities of the Parent *versus* the *ΔerpΔpδ* Strains

**Figure 5:**
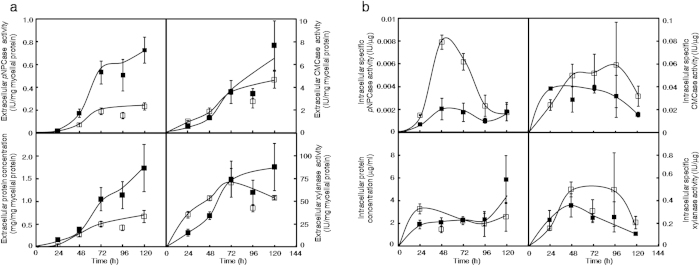
Comparison of the Protein Secretion Capabilities of the Parent and *ΔerpΔpδ* Strains. Panel **a**, comparison of extracellular protein concentrations and enzymatic activities between the parent and *ΔerpΔpδ* strains. Closed square, parent strain; open square, *ΔerpΔpδ* strain. Panel **b**, comparison of intracellular protein concentrations and specific enzymatic activities between the parent and *ΔerpΔpδ* strains. Closed square, parent strain; open square, *ΔerpΔpδ* strain. Error bars indicate the standard deviation calculated from three biological experiments.

Equal quantities of precultured mycelia were inoculated into liquid media containing 2% Avicel to compare the relative protein secretion capabilities of the parent and *ΔerpΔpδ* strains. CMCase, *p*NPCase and xylanase activities represent endoglucanase, cellobiohydrolase and xylanase activities, respectively. The results for the extracellular enzymatic activities normalised to intracellular proteins are shown in Fig. 5a. For the *ΔerpΔpδ* strain, extracellular *p*NPCase activity significantly decreased after 48 hours of cultivation (P < 0.05, n = 3). The extracellular protein concentration significantly decreased in the *ΔerpΔpδ* strain after 72 hours following inoculation (P < 0.05, n = 3). However, CMCase and xylanase activities were not significantly changed in the *ΔerpΔpδ* strain. Intracellular specific enzymatic activities were also detected. As shown in Fig. 5b, there were no significant differences between specific CMCase and xylanase activities. However, specific *p*NPCase activity significantly increased at 24, 48, and 96 hours after inoculation (P < 0.05, n = 3). Therefore, protein secretion declined in the *ΔerpΔpδ* strain. This suggests that the p24 heterodimer is likely involved in protein transport, particularly in the transport of cellobiohydrolase.

### Intracellular Proteomic Analysis after p24 Heterodimer Disruption in *P. oxalicum*

**Figure 6:**
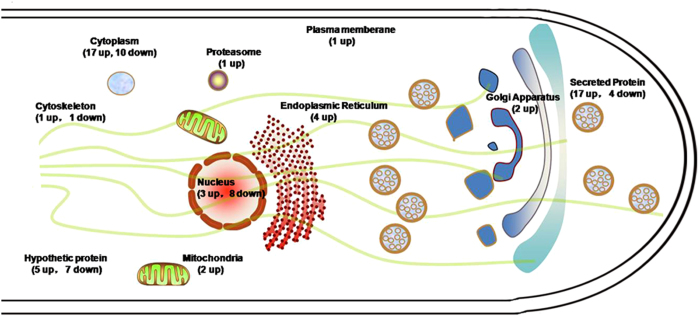
Subcellular Distribution of Significantly Changed Proteins in Response to *erp* and *pδ* Deletions in *P. oxalicum.*

Intracellular proteomic analysis was performed using label-free liquid chromatography mass spectrometry to determine the intracellular response of *P. oxalicum* under cellulase-inducing conditions after p24 heterodimer disruption. Eighty-three proteins were significantly altered, and detailed information concerning these proteins is shown in Supplementary Information Table S1 online. According to published studies of homologues of these proteins^31^,^32^, they were broadly distributed in the nuclear lumen, ER, Golgi-like apparatus, mitochondria, cytoskeleton, proteasome, and cytoplasm (Fig. 6).

The *ΔerpΔpδ* strain had 53 proteins that were significantly increased in concentration; of these, 17 were secreted proteins. All of the cellobiohydrolase proteins (PDE_07124, PDE_05445, and PDE_07945) in *P. oxalicum* were significantly increased. PDE_02805, PDE_05263, PDE_02536, PDE_03112, and PDE_04618, which were predicted to be GPI-anchored proteins, were significantly upregulated in the *ΔerpΔpδ* strain as well^33^.

There were 30 significantly decreased proteins in the *ΔerpΔpδ* strain. Five proteins (PDE_09561, PDE_04823, PDE_02141, PDE_01235 and PDE_02162) were related to biosynthesis. Four proteins (PDE_02839, PDE_06220, PDE_02391, and PDE_09925) were involved in DNA replication. Two rRNA-related proteins (PDE_07738 and PDE_06767) were significantly downregulated in the *ΔerpΔpδ* strain.

## Discussion

The protein secretory pathway is important in many organisms. This pathway is comprised by membrane-bound compartments, and the p24 proteins are integral membrane components^34^. Previous studies have investigated functions of the p24 proteins in *Saccharomyces* spp., *Drosophila*, and mammalian cells. Erp in filamentous fungi is involved in cellulase secretion. However, the functions of other p24 proteins (single or heterodimers) in filamentous fungi remain unknown. Thus, we investigated the function of the p24 heterodimer consisting of Erp and Pδ in *P. oxalicum*.

The p24 proteins affect physiological pathways by forming functional oligomers^28^,^35^. The coiled-coil regions in yeast are responsible for the assembly of p24 hetero-oligomers^24^. These coiled-coil regions were also found in Erp and Pδ (Fig. 1b). The BiFC assay suggested the interaction of these proteins (Fig. 2). Therefore, Erp and Pδ possibly form a heterodimer or a heterocomplex in *P. oxalicum*.

Compared with *P. oxalicum Δerp*, decreased sporulation and altered lateral branching were noted in *ΔerpΔpδ*. This suggested the disruption of more cellular physiological pathways in *ΔerpΔpδ,* which possibly implies that more functional oligomers are formed by these proteins.

Branching in filamentous fungi enhances nutritional assimilation by increasing colony surface and facilitates the exchange of important signals by triggering hyphal fusion^36^. Tip and lateral branching are two types of branching. Lateral branches are oriented perpendicular to the main hyphae of filamentous fungi^37^. However, the *ΔerpΔpδ* strain exhibited altered lateral branching but normal tip branching (Fig. 4a). Tip and lateral branching can involve different morphogenesis and regulatory mechanisms^38^. The *pod-4* and *pod-8* defective mutants in *Neurospora crassa* exhibit wild type tip growth, but the branching in their subapical regions is contorted^37^. Lateral branching consists of four steps. The last step is lateral branching maturation, which depends on microtubules^37^. The abnormal lateral branching patterns in the *ΔerpΔpδ* strain may be attributed to the disruption of specific proteins that locally modify microtubules or other proteins that direct lateral branch growth. The eukaryotic-type Ser/Thr protein kinase AfsK in the filamentous bacterium *Streptomyces* phosphorylates DivIVA, a polar recruitment factor or cell polarity determinant, to regulate the pattern of lateral branch formation^39^,^40^. The specific proteins that direct lateral branch growth may be dislocated in the *ΔerpΔpδ* strain and may change the pattern of lateral branch development.

Previous reports have shown that *erp* deletion causes secretion stress in filamentous fungi^27^. Seventeen of the 53 significantly upregulated intracellular proteins in the *ΔerpΔpδ* strain were predicted to be secreted proteins. All of the cellobiohydrolase proteins were upregulated in *ΔerpΔpδ* mycelia. The cellobiohydrolase content of Cel7A-2 (PDE_07945) comprises approximately 16% of total extracellular proteins under inducing conditions in *P. oxalicum* 114-2^6^. It was upregulated by 3.59-fold in the *ΔerpΔpδ* intracellular proteome. Furthermore, in *P. oxalicum,* extracellular *p*NPCase activity was significantly decreased and intracellular specific *p*NPCase activity was significantly increased. These results were consistent with each other. Therefore, it is likely that inefficient protein transport causes a “traffic jam” and subsequent secretion stress in the *ΔerpΔpδ* strain. Furthermore, we suggest that the p24 heterodimer participates in protein transport, particularly cellobiohydrolase.

The p24 proteins participate in the transport of GPI-anchored proteins in *S. cerevisiae* or mammalian cells^41^,^42^. Some GPI-anchored proteins (PDE_02805, PDE_05263, PDE_02536, PDE_03112, and PDE_04618) were upregulated in *ΔerpΔpδ.* This result indicates that the mediating effects of the p24 proteins on GPI-anchored protein transport are conserved in filamentous fungi.

The *Aspergillus niger* homolog of PDE_06282, which was upregulated in the *ΔerpΔpδ* strain, is involved in ER quality control^43^. A BlastP search against the *Saccharomyces* Genome Database yielded YOS9, albeit with low sequence identity, which recognises N-linked glycans to discriminate misfolded proteins. YOS9 helps degrade misfolded proteins in endoplasmic reticulum-associated degradation (ERAD)^43^,^44^,^45^. PDE_02603, a candidate 26S proteasome regulatory subunit, was upregulated by 5.63-fold. The 26S proteasome degrades ubiquitinated targets and is also involved in ERAD^46^. PDE_04048 and PDE_05480 are aminopeptidases that digest amino acids from the N-terminus of peptides in the intracellular environment. We propose that the *ΔerpΔpδ* strain attempts to degrade proteins that clutter the endomembrane system in response to secretion stress. These proteins are probably extracellularly secreted in the parent strain.

Four DNA replication-related proteins in the nucleus (PDE_02839, PDE_06220, PDE_02391, and PDE_09925) were significantly decreased in *ΔerpΔpδ*. We considered these proteins to be growth-related proteins because DNA replication is related to cell cycle progression. This result was consistent with the decrease in colony diameter of the *ΔerpΔpδ* strain grown on wheat bran plates. Some rRNA-related genes were also significantly changed. One gene (PDE_08726) was increased and two genes (PDE_07738, PDE_06767) were decreased in the *ΔerpΔpδ* strain. PDE_04575, whose homolog in yeast is PUF4, was upregulated in the *ΔerpΔpδ* strain. PUF4 represses rRNA biogenesis gene expression by binding to the 3′-UTR sequence^47^. Some biosynthesis-related genes (PDE_02141, PDE_01235, PDE_04823, PDE_02162, and PDE_09561) were also downregulated in the *ΔerpΔpδ* strain. This result may be attributed to the notion that p24 heterodimer disruption stresses *P. oxalicum* 114-2 secretion and then forces cells to reprogram gene expression to alleviate this stress. DNA replication and biosynthesis-related genes are repressed for energy conservation, and stress-reducing genes are activated for survival. The DNA replication and biosynthesis-related genes in yeast are also generally repressed by stress^48^,^49^. This result is consistent with the observations in *P. oxalicum*.

*S. cerevisiae* and *P. oxalicum* are both fungi, but *S. cerevisiae* and *P. oxalicum* p24 deletion strains exhibit significantly different morphogenesis and cargo transport. The *S. cerevisiae* p24*Δ*8 strain has minor defects in cargo transport and grows identical to the wild type, but the *P. oxalicum ΔerpΔpδ* strain exhibits major defects in morphogenesis and cargo transport^50^. These results reflect the divergence of p24 protein functions between species.

Four p24 homologs were identified in *P. oxalicum* 114-2 through bidirectional BlastP best-hit search. Phylogenetic tree analysis revealed that these four proteins belong to four different p24 subfamilies. From this group, Erp and Pδ were chosen for further study. The two p24 proteins were likely to form a p24 heterodimer *in vivo*. The p24 heterodimer was found to participate in important morphogenetic events, including sporulation, hyphal growth, and lateral branching. Protein secretion ability declined in the *ΔerpΔpδ* strain. Global intracellular proteomic analysis revealed that the *ΔerpΔpδ* strain was under secretion stress, and removal of proteins jammed in the endomembrane system occurred as a result. These results suggest that the p24 heterodimer participates in morphogenesis and protein transport. Compared with *Δerp*, more cellular physiological pathways were disrupted in *ΔerpΔpδ*, which possibly implies that more functional oligomers were formed by these proteins. These findings provide insights into the secretion pathways of filamentous fungi.

## Methods

### Genetic Construction of Strains

*P. oxalicum* 114-2 *Δpku70*^51^ was used in this study as the parent strain. The *P. oxalicum* p24 double deletion strains were obtained by transforming knockout cassettes into recipient strains. The GenBank accession numbers for Erp, Pδ, PDE_03657 and PDE_00613 in NCBI were EPS34507.1, EPS33374.1, EPS28711.1 and EPS25679.1, respectively. The strains for the BiFC assay were constructed as previously described^52^. Detailed information on these strains is listed in Supplementary Information Table S2 online.

Individual *P. oxalicum* strains were transformed as previously described^51^. The media for transformation contained 350 μg/ml hygromycin B, 0.2 μg/ml pyrithiamine, and/or 12 μg/ml basta. The transformants with cassettes integrated at the desired loci were identified by Southern blotting (DIG High Prime labelling kit, Roche Applied Science). Equal amounts of intracellular proteins from six double-deletion strains were electrophoretically resolved in a 15% SDS-PAGE separating gel containing 6 M urea for SDS-PAGE analysis.

### Sequence Alignment and Phylogenetic Tree Construction

The amino acid sequences of the p24 proteins in Supplementary Information Fig. S1 were aligned using Clustal X2.0. The phylogenetic tree in Fig. 1a was constructed using MEGA 5.05 via the neighbour-joining method. Bootstrap values calculated from 1000 trees are shown at each node. The bar indicates an evolutionary distance of 0.2. The NCBI database accession numbers of the proteins in Fig. 1a are shown in Supplementary Information S1 online.

### BiFC Assay

Spores were collected from 5-day-old strains grown on wheat bran plates at 30 °C. The spores were inoculated into submerged media containing 2% glucose and minimal media (MM) solution at 30 °C for 48 h. The composition of the MM solution was as follows: 0.5% (NH_4_)_2_SO_4_, 0.06% MgSO_4_, 1.5% KH_2_PO_4_, 0.08% CaCl_2_, 0.00005% FeSO_4_·7H_2_O, 0.00016% MnSO_4_·H_2_O, 0.00014% ZnSO_4_·7H_2_O and 0.00002% CoCl_2_. The hyphae were observed under a LSM 780 Laser Scanning Confocal Microscope (Carl Zeiss SAS, Jena, Germany). Excitation was set to 514 nm for EYFP. Emission was selected by bandpass filters BP 530 to 600. The captured images were analysed using the Zen 2012 Blue Edition software.

### Sporulation Assay

Approximately 1 × 10^6^ spores from the parent and *ΔerpΔpδ* strains were inoculated onto wheat bran plates for 3 d. The spores were washed with solutions containing 0.9% NaCl and 0.5% Tween-80 and then counted using a haemocytometer. Six technical replicates for each biological replicate and three biological replicates were performed. Standard errors were calculated from three biological replicates. Statistical analysis was performed with two-tailed Student’s *t*-tests, and P < 0.05 was considered indicative of statistical significance.

The induction of sporulation in submerged cultivation was carried out as previously described^53^ with modification. Approximately 1 × 10^7^ spores were inoculated into medium A (medium A contained 5% glucose, 0.1% KH_2_PO_4_, 0.05% MgSO_4_·7H_2_O, 0.23% KNO_3_, 0.000025% ZnSO_4_·7H_2_O, 0.00002% FeSO_4_·7H_2_O, 0.000004% (NH_4_)_6_MoO_24_·4H_2_O, 0.0000038% CuSO_4_·5H_2_O and 0.0000024% MnSO_4_) and cultivated at 25 °C and 200 rpm for 24 h in the dark. The mycelia were subsequently filtered, washed and transferred into 50 ml of medium B (medium B was the same as medium A without the nitrogen source). The cultures were incubated at 25 °C and 200 rpm for 144 h in the dark. Fifty millilitres of the cultures were equally divided into two fractions. The first fraction was filtered through Whatman no. 1 filter paper and ground into powder in liquid nitrogen. The powder was collected, dissolved in 2 ml of 1 M NaOH solution and centrifuged at 12000 × *g* for 10 min at 4 °C. The supernatants were extracted to test intracellular protein concentrations by the Lowry method^54^. The second fraction was filtered through lens wiping paper after the addition of 0.2 ml of Tween-20. The spores were counted using a haemocytometer. Five biological replicates were carried out. The standard errors were calculated from five biological replicates. Statistical analysis was performed with two-tailed Student’s *t*-tests, and P < 0.05 was considered indicative of statistical significance.

### Phenotypic Observation and Microscopic Examination of Lateral Branching Patterns

For microscopic observation, 1 × 10^3^ spores were inoculated onto agar media containing 2% glucose and MM. The media were placed on glass slides and covered with cover slips. The spores were incubated at 30 °C for 48 h and then observed under a microscope (Nikon Eclipse E100, 400-fold magnification). Images were captured using a Nikon D5000. The angles between the main hyphae and branches were measured using ImageJ software. Three biological replicates were carried out. One hundred angles were measured for each of the biological replicates. Standard errors were calculated from three biological experiments. Statistical analysis was performed using two-tailed Student’s *t*-tests, and P < 0.05 was considered indicative of statistical significance.

### Assessment of Protein Secretion Capabilities of Deletion Strains

Approximately 1 × 10^7^ spores were inoculated into liquid media containing 2% glucose, 0.5% tryptone and MM. They were cultivated at 30 °C and 200 rpm for 48 h. The cultures were filtered and washed with 200 ml of a solution containing 0.9% NaCl and 0.5% Tween-80. Approximate 5 g of mycelia were transferred into 150 ml of liquid media containing 2% Avicel, MM and 0.5% urea. The mycelia were incubated at 30 °C and 200 rpm for 120 h. Ten millilitres of each sample was drawn every 24 h and centrifuged at 12000 × *g* for 10 min at 4 °C. The supernatants were extracted to analyse extracellular enzyme activities and protein concentrations. The sediments were used for the preparation of mycelial proteins as previously described^55^.

For the measurement of intracellular specific enzymatic activities, the cultivation of fungi was performed as described above. Ten millilitres of cultures were drawn every 24 h after inoculation. They were filtered through Whatman no. 1 filter paper and ground into powder in liquid nitrogen. The powder was collected and dissolved in 3 ml of buffer containing 50 mM acetate (pH = 4.8) and centrifuged at 12000 × *g* for 10 min at 4 °C. The supernatants were collected to test intracellular protein concentrations and enzyme activities.

The extracellular protein concentration was measured by the Bradford assay^56^. Tests for CMCase, *p*NPCase, and xylanase activities and mycelial protein content were performed as described elsewhere^55^,^57^. Statistical analysis was performed with two-tailed Student’s *t*-tests, and P < 0.05 was considered indicative of statistical significance.

### Quantitative Intracellular Proteomics Comparison of Parent and Deletion Strains using Label-Free Chromatography Mass Spectrometry

Approximately 1 × 10^7^ spores from the parent and *ΔerpΔpδ* strains were inoculated into liquid media containing 2% glucose and MM. The spores were incubated at 200 rpm and 30 °C for 48 h. Mycelia were harvested and washed twice with a solution containing 0.9% NaCl and 0.5% Tween-80. Approximately 5 g of mycelia were transferred into liquid media containing 2% Avicel, MM and 0.5% urea. The spores were incubated at 200 rpm and 30 °C for 48 h. The cultures were filtered, washed with 200 ml of a solution containing 0.9% NaCl and 0.5% Tween-80 and then ground into powder in liquid nitrogen. The powder was dissolved into 30 ml of buffer containing 100 mM ammonium bicarbonate (pH 7.4) and then centrifuged at 35000 × *g* for 1 h at 4 °C. The supernatant was extracted and concentrated into 1 ml using a 3-kDa Pall Nanosep® centrifugal device. Protein content was measured using the Bradford assay^53^.

An equal amount of protein was reduced using 10 mM dithiothreitol at 50 °C for 45 min, and the proteins were alkylated using 55 mM iodoacetamide at room temperature for 45 min in the dark. The samples were digested using trypsin (1:20 w/w) at 37 °C for 24 h and then purified using ZipTips (Millipore, Zug, Switzerland). The peptides were vacuum-dried into powder and dissolved in 0.1% formic acid. The peptides were separated using a nano-HPLC (PROXEON, Thermo) supplied with reversed-phase chromatography (PROXEON, 15 cm × 75 μm, 3 μm/100 Å pore size, LC Packings). Approximately 0.6 μg of each sample was loaded and eluted using a nonlinear 90-min gradient of solutions A (0.1% formic acid in water) and B (0.1% formic acid in 100% acetonitrile). The flow rate was set at 250 nL/min. The gradient setting was between 2% and 5% B for 0 to 5 min and between 5% and 35% B for 5 to 95 min. The eluted peptides were then loaded into a linear quadrupole ion trap orbitrap mass spectrometer (LTQ Orbitrap Elite, ThermoFisher, Bremen, Germany) with a nanoelectrospray ion source. Full-scan MS survey spectra (from m/z 350 to 1800) with a resolution of R = 60,000 were acquired at m/z 400 using the orbitrap after accumulating 1,000,000 ions. The twenty most intense ions were isolated and fragmented by collision-induced dissociation at the target value of 10,000 ions. The electrospray voltage was set to 2.2 kV, and the dynamic exclusion duration was set to 90 s. Normalised collision energy was set to 35%. The activation Q-value was 0.25. The activation time was 10 ms. The ion selection threshold was 1000 counts for MS/MS. Three technical replicates for each sample were processed.

The acquired spectra were imported into Progenesis LC-MS software (version 4.1, Nonlinear Dynamics) for label-free quantification. One sample was set as the reference, and the retention times of two samples were automatically aligned to create the maximal overlap. Peptides with +2, +3, +4, +5, and +6 charges were used for further analysis. The abundance of the remaining features in each sample was used to calculate a normalisation factor and correct for experimental variation. Only unique peptides with a Mascot score >30 and P < 0.01 were quantified, and the total cumulative abundance was calculated by summing the abundance of the peptides assigned to each protein. The means within each group were used to calculate the fold change, and the P-value of each protein was calculated by two-way ANOVA analysis using the sum of the normalised abundance across all runs. The quantitation files were exported and searched against Mascot (version 2.4) in the *P. oxalicum* 114-2 genome database. The search parameters were as follows: enzyme, trypsin; ion score cut-off, 30; significance threshold, P < 0.01; peptide mass tolerance, 10 ppm; and fragment mass tolerance, 0.8 kDa. Two missed cleavages were allowed, cysteine carbamidomethylation was set as a fixed modification, and methionine oxidation was set as a variable modification. The peptides were assigned to proteins, and the files were re-imported into Progenesis LC-MS software. The significantly changed proteins exhibited fold change >1.5 or <1.5 and P < 0.01. Functional annotation of the significantly changed proteins was obtained from the *P. oxalicum* 114-2 genome database hosted on NCBI^6^. The proteins annotated as hypothetical proteins were re-BLASTed against the *Saccharomyces* Genome Database or the UniProt database (sequence identity >30%, E-value cut-off of 1E -5).

## Additional Information

**How to cite this article**: Wang, F. *et al.* Function of a p24 heterodimer in morphogenesis and protein transport in *Penicillium oxalicum. Sci. Rep.* **5**, 11875; doi: 10.1038/srep11875 (2015).

## Supplementary Material

Supplementary Information
