# Supplementary material for: Function of a p24 Heterodimer in Morphogenesis and Protein Transport in Penicillium oxalicum
Source: Sci Rep. 2015 Jul 7;5:11875. doi: 10.1038/srep11875 (PMC4493713; doi:10.1038/srep11875)
Supplement: Supplementary Information [file srep11875-s1.pdf]

Function of a p24 heterodimer in morphogenesis and protein transport in *Penicillium oxalicum*

Fangzhong Wang [1], Kuimei Liu [1], Lijuan Han [1], Baojie Jiang [1], Mingyu Wang [1], Xu Fang [1,*]

[1] State Key Laboratory of Microbial Technology, School of Life Science, Shandong University, Jinan, Shandong, China

[*] To whom correspondence is addressed. E-mail: fangxu@sdu.edu.cn

Tel: +86-531-88364004, Fax: +86-531-88364363

Supplementary Information

Fig. S1

Sequence alignment of p24 homologs in *P. oxalicum* 114-2 and *S.cerevisiae.*

The same amino acid residues are shaded.

Fig. S2

SDS-PAGE analysis of intracellular proteins of p24 deletion strains grown in liquid media containing 2% Avicel, MM and 0.5% urea.

M: protein marker; 1: parent strain; 2: *P. oxalicum* 114-2 *ΔpδΔPDE_03657*;

3: *P. oxalicum* 114-2 *ΔerpΔpδ*; 4: *P. oxalicum* 114-2 *ΔpδΔPDE_00613*;

5: parent strain; 6: *P. oxalicum* 114-2 *ΔPDE_00613ΔPDE_03657*;

7: *P. oxalicum* 114-2 *ΔerpΔPDE_03657*; 8: *P. oxalicum* 114-2 *ΔerpΔPDE_00613*.

Fig. S3

Deletion of the *pδ* gene was confirmed by Southern blotting.

Panel a, schematic diagram of Southern blotting. Panel b, Southern blotting analysis of *pδ* deletion. M: 1 kb marker; 1: *P. oxalicum Δerp* strain; 2: *P. oxalicum ΔerpΔpδ* strain. Arrows are indicated the predicted band sizes of each strain.

Table S1 Proteins were significantly increased or decreased in *ΔerpΔpδ* strain.

* were predicted as GPI-anchor proteins

Table S2 *P. oxalicum* strains that were used in this study.

S1 Protein accession numbers in the phylogenetic tree in Fig. 1a.

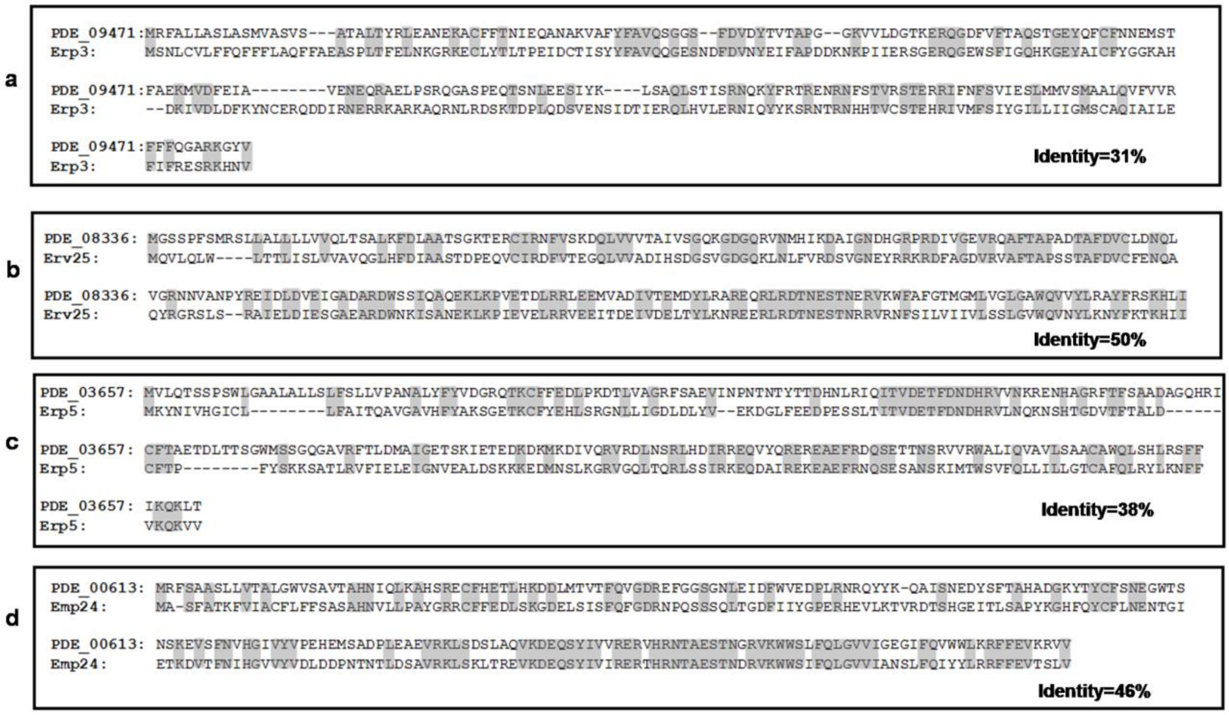

Fig. S1 Sequence alignment of p24 homologs in *P. oxalicum* 114-2 and *S. cerevisiae.*

The same amino acid residues are shaded.

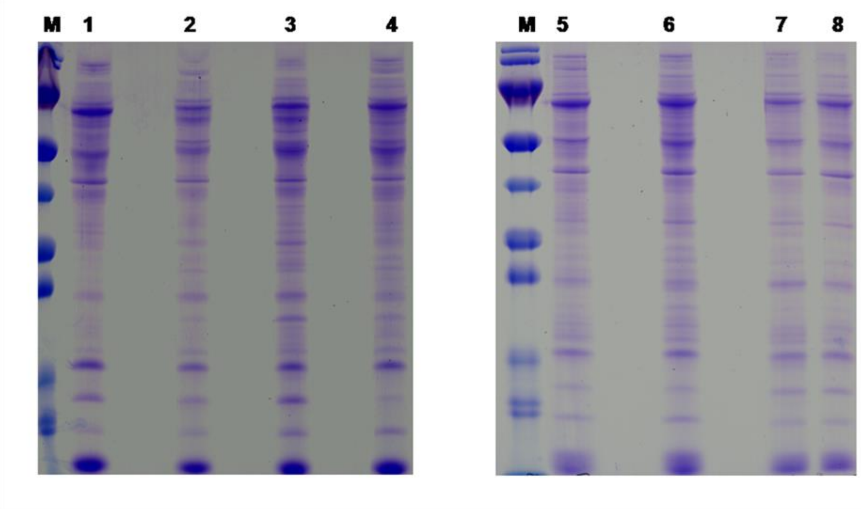

Fig. S2 SDS-PAGE analysis of intracellular proteins of p24 deletion strains grown in liquid media containing 2% Avicel, MM and 0.5% urea.

M: protein marker; 1: parent strain; 2: *P. oxalicum* 114-2 *ΔpδΔPDE_03657*; 3: *P. oxalicum* 114-2 *ΔerpΔpδ*; 4: *P. oxalicum* 114-2 *ΔpδΔPDE_00613*; 5: parent strain; 6: *P. oxalicum* 114-2 *ΔPDE_00613ΔPDE_03657*;

7: *P. oxalicum* 114-2 *ΔerpΔPDE_03657*; 8: *P. oxalicum* 114-2 *ΔerpΔPDE_00613*.

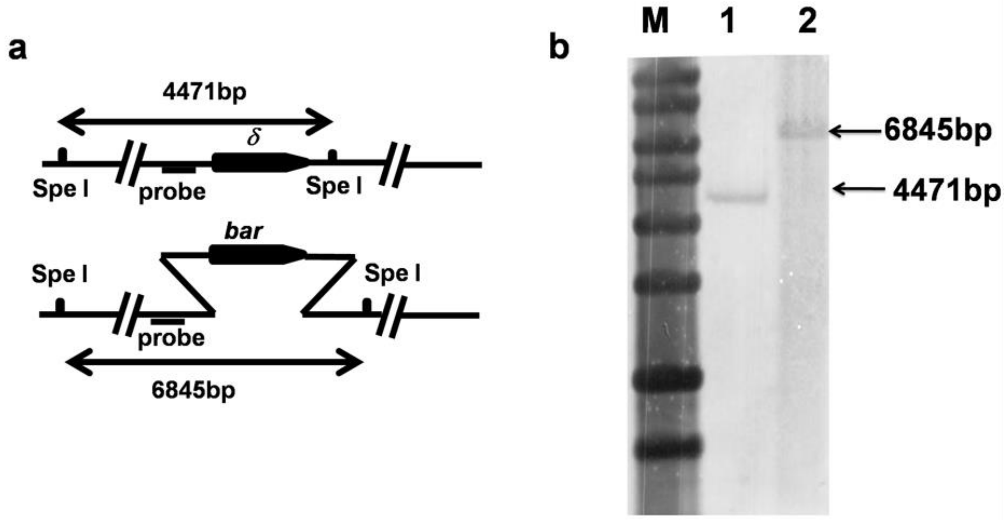

Fig. S3 Deletion of the *pδ* gene was confirmed by Southern blotting.

Panel a, schematic diagram of Southern blotting. Panel b, Southern blotting analysis of *pδ* deletion. M: 1 kb marker; 1: *P. oxalicum Δerp* strain; 2: *P. oxalicum ΔerpΔpδ* strain. Arrows are indicated the predicted band sizes of each strain.

Table S1 Proteins were significantly increased or decreased in *ΔerpΔpδ* strain.

* were predicted as GPI-anchor proteins

Table S 1.1 Cytoplasm (Secreted protein)

Gene name	Protein name	Fold change	P value	Predicted protein function
PDE_09285	α-L-rhamnopyranohydrolase, GH28	positive infinity	1.24e-04	Rhamnogalacturonan hydrolysis
PDE_02805*	Hypothetical protein	151.66	2.21e-04	Unknown
PDE_07124	Cellobiohydrolase, GH6	64.4	2.23e-03	Cellulose hydrolysis
PDE_07936	Metallo peptidase	29.53	1.43e-03	Polypeptide hydrolysis
PDE_09226	Endo-β-1,4-glucanase, GH5	14.45	3.97e-04	Cellulose hydrolysis
PDE_07929	Endo-β-1,4-glucanase, GH7	11.29	1.63e-03	Cellulose hydrolysis
PDE_05263*	Chitin glucanosyltransferase, GH16	9.09	2.63e-03	The transfer of chitin to β-1, 3- and β-1, 6-glucans
PDE_05445	Cellobiohydrolase, GH7	8.83	9.68e-05	Cellulose hydrolysis
PDE_07897	α-L-arabinofuranosidase, GH62	8.79	2.61e-04	Arabinoxylan hydrolysis
PDE_06439	Endo-β-1,4-glucanase, GH12	7.38	4.02e-05	Cellulose hydrolysis
PDE_06649	Feruloyl esterase, CE1	6.91	9.12e-03	Arabinoxylan hydrolysis

PDE_02536*	Hypothetical protein	3.96	4.64e-03	Unknown
PDE_07945	Cellobiohydrolase, GH7	3.59	1.00e-02	Cellulose hydrolysis
PDE_08094	Endo-β-1,4-xylanase, GH10	3.43	3.32e-04	Xylan hydrolysis
PDE_03112*	Exo-β-1,3-glucanase, GH17	2.83	7.96e-03	β-1, 3-glucans hydrolysis
PDE_08122	Chitinase, GH18	2.61	5.09e-03	Chitin hydrolysis
PDE_00500	Non-hemolytic phospholipase C	1.7	8.38e-03	Phosphatidylcholine and phosphatidylserine hydrolysis
PDE_09417	Glucoamylase, GH15	-1.97	1.84e-03	α-1, 4-glucose hydrolysis
PDE_00615	β-1,3-glucanosyltransferase, GH17	-2	1.00e-02	Transferring the newly generated reducing end to the non-reducing end of another β-1, 3-glucan molecule, and generating a new β-1, 6-linkage
PDE_06770	Hypothetical protein	-2.03	8.77e-03	Unknown
PDE_03966	α-glucosidase, GH31	-3.18	2.03e-04	Maltose and other small maltooligosaccharides hydrolysis

Table S 1.2 Cytoplasm

**Gene name**	**Protein name**	**Fold change**	**P value**	**Predicted protein function**
PDE_02929	D-xylose reductase	positive infinity	2.95e-03	Reducing D-xylose into xylitol

PDE_01713	Formamidase	13.19	4.23e-05	Formamide hydrolysis
PDE_02372	Coproporphyrinogen III oxidase	12.66	1.00e-02	The heme biosynthesis
PDE_09398	Transaldolase	11.47	1.80e-03	Pentose-phosphate pathway
PDE_03821	Mannitol-1-phosphate 5-dehydrogenase	9.1	5.96e-03	Fructose and mannose metabolism
PDE_08726	Ribosome biogenesis protein	7.53	1.07e-03	20S pre-rRNA processing; 40S ribosomal subunit synthesis
PDE_05515	β-1,3-glucanase	7.44	5.88e-05	Hydrolysis of 1, 3- linkages in β-glucans
PDE_02988	PLC-like phosphodiesterase	7.28	9.09e-03	Phosphatidylinositol metabolism
PDE_04575	Member of the PUF protein family	6.25	3.25e-04	Binding mRNAs encoding nucleolar ribosomal RNA-processing factors
PDE_04048	X-Pro aminopeptidase	4.42	2.13e-04	Peptidase hydrolysis
PDE_02117	Guanyl-specific ribonuclease	4.23	1.41e-04	Endoribonuclease activity
PDE_04364	GDSL esterase/lipase	3.69	6.84e-03	Easter bonds hydrolysis
PDE_01218	ABC transporter	3.39	1.44e-03	Translocation of a variety of compounds across transmembrane
PDE_04898	Chitinase	2.51	2.15e-03	Chitin hydrolysis
PDE_05480	Aminopeptidase	2.31	5.30e-03	Peptidase hydrolysis

PDE_03243	Esterase/lipase	1.8	1.00e-02	Easter bonds hydrolysis
PDE_04618*	β-1,3-glucanosyltransferase, GH72	1.51	7.97e-03	Transferring the newly generated reducing end to the non-reducing end of another β-1, 3-glucan molecule and elongating the β-1, 3-linkage
PDE_04468	α-1,6-mannanase	-1.55	1.14e-03	Random hydrolysis of α-1, 6-mannosidic linkages in unbranched 1, 6-mannans
PDE_02839	DNA replication licensing factor Mcm3	-1.72	8.61e-03	DNA replication; Component of the Mcm2-7 hexameric helicase complex
PDE_08209	Dienelactone hydrolase family protein	-2.03	8.77e-03	Aromatic compounds degradation
PDE_09561	Lanosterol synthase	-2.9	1.96e-03	Ergosterol biosynthesis
PDE_06127	Ribonucleoside-diphosphate reductase subunit M1	-4.56	3.83e-03	Catalyzing rate-limiting step in dNTP synthesis
PDE_04823	Hydroxymethylglutaryl-CoA synthase	-4.69	6.03e-03	Mevalonate biosynthesis
PDE_02141	Polyketide synthase	-5.9	1.08e-04	Polyketides biosynthesis
PDE_01235	Polyketide synthase	-14.79	6.41e-05	Polyketides biosynthesis
PDE_02162	The dioxygenase family protein	-22.32	7.55e-04	Betalain biosynthesis
PDE_01429	Cytochrome P450 family protein	-319.88	1.92e-03	Oxidoreductase activity

Table S 1.3 proteasome

Gene name	Protein name	Fold change	P value	Predicted protein function
PDE_02603	26S proteasome regulatory subunit	5.63	4.98e-04	Degrading proteins that have been ubiquinated

Table S 1.4 Nucleus

Gene name	Protein name	Fold change	P value	Predicted protein function
PDE_08726	Ribosome biogenesis protein	7.53	1.07e-03	20S pre-rRNA processing; 40S ribosomal subunit synthesis
PDE_06111	DNA mismatch repair protein	3.59	4.31e-04	DNA mismatch repair
PDE_05989	Kinesin family member 11	3.58	1.28e-03	Spindle assembly and chromosome segregation
PDE_02839	DNA replication licensing factor Mcm3	-1.72	8.61e-03	Protein involved in DNA replication; Component of the Mcm2-7 hexameric helicase complex
PDE_06220	DNA polymerase epsilon subunit 2	-2.04	5.31e-03	The maintenance of fidelity of chromosomal replication
PDE_07239	Quinate repressor protein	-2.39	1.00e-02	Transcription of the quinate utilization pathway genes
PDE_02391	Chromosome segregation protein	-2.48	1.45e-04	Sister chromatid cohesion and linking DNA synthesis to sister chromatid cohesion
PDE_07738	rRNA biogenesis protein	-3.33	1.99e-04	18S and 5.8S rRNA biogenesis

PDE_05592	DEAD/DEAH box helicase	-4.39	5.95e-03	Nuclear RNA processing and degradation
PDE_06767	Nucleolar protein	-5.42	1.00e-02	35S pre-RNA processing; 40S ribosomal subunit biogenesis
PDE_09925	Nuclear condensin complex subunit 3	-5.9	5.95e-05	The establishment and maintenance of chromosome condensation; the segregation and chromatin binding of the condensin complex

Table S 1.5 Cytoskeleton

Gene name	Protein name	Fold change	P value	Predicted protein function
PDE_08653	Dynactin, 150 kDa	2.47	5.48e-03	Dynactin is a multisubunit complex required for cytoplasmic dynein to efficiently transport vesicles along microtubules.
PDE_00169	Arp2/3 complex subunit 2	-12.02	1.63e-05	The regulation of actin polymerization and mediating the formation of branched actin networks

Table S 1.6 Golgi-like apparatus

Gene name	Protein name	Fold change	P value	Predicted protein function
PDE_04430	Guanyl-nucleotide exchange factor	2.45	1.24e-03	Arl1p activation and vesicular transport
PDE_03529	Aminophospholipid translocase	2.15	8.94e-03	Endocytosis and vacuolar biogenesis

Table S 1.7 Mitochondria

Gene name	Protein name	Fold change	P value	Predicted protein function
PDE_09399	Superoxide dismutase [Cu-Zn]	6.28	4.15e-04	Detoxifying superoxide
PDE_05733	3-hydroxybutyryl-CoA dehydrogenase	5.53	5.78e-03	Catalyzing the NAD-dependent dehydration of β-hydroxybutyryl-CoA to acetoacetyl-CoA

Table S 1.8 Endoplasmic reticulum

Gene name	Protein name	Fold change	P value	Predicted protein function
PDE_08512	Alkaline ceramidase	7	7.37e-03	Sphingolipid ceramide hydrolysis
PDE_07729	Phosphatidylethanolamine N-methyltransferase	6.06	4.82e-04	The conversion of phosphatidylethanolamine to phosphatidylcholine
PDE_03488	Fatty acid transporter protein	5.89	7.55e-04	Long chain fatty acids transport
PDE_06282	ER quality control lectin	1.89	2.12e-03	The bridging protein between the machinery that extracts misfolded proteins from the ER lumen and the machinery involved in their retro-translocation across the ER-membrane

Table S 1.9 Plasma membrane

Gene name	Protein name	Fold change	P value	Predicted protein function
PDE_04996	Putative sensor/transporter protein	2.55	1.13e-03	Cell wall biogenesis

Table S 1.10 Hypothetical protein

Gene name	Protein name	Fold change	P value	Predicted protein function
PDE_02805*	Hypothetical protein	151.66	2.21e-04	Unknown
PDE_02536*	Hypothetical protein	3.96	4.64e-03	Unknown
PDE_08596	Hypothetical protein	2.03	2.81e-03	Unknown
PDE_05474	Hypothetical protein	1.73	1.49e-03	Unknown
PDE_06770	Hypothetical protein	6.49	4.14e-03	Unknown
PDE_07440	Hypothetical protein	-2.65	8.42e-03	Unknown
PDE_09712	Hypothetical protein	-3.09	1.00e-02	Unknown
PDE_05911	Hypothetical protein	-3.12	9.30e-04	Unknown
PDE_06953	Hypothetical protein	-3.41	4.40e-03	Unknown
PDE_00315	Hypothetical protein	-5.64	5.82e-04	Unknown
PDE_03744	Hypothetical protein	-13.12	1.46e-04	Unknown

PDE_06675	Hypothetical protein	minus infinity	6.74e-04	Unknown

Table S2 *P. oxalicum* strains that were used in this study.

strains	Genotype	Source
P. oxalicum 114-2	Wildtype	Reference 6
P. oxalicum 114-2 *Δpku70*	*Δpku70*::*hph*	Reference 51
P. oxalicum 114-2 *Δerp*	*Δpku70*::*hph*; *Δerp*::*ptra*	Reference 27
P. oxalicum 114-2 *ΔerpΔpδ*	*Δpku70*::*hph*; *Δerp*::*ptra*; *Δpδ*::*bar*	This study
P. oxalicum 114-2 *ΔerpΔPDE_00613*	*Δpku70*::*hph*; *Δerp*::*ptra*; *ΔPDE_00613*::*bar*	This study
P. oxalicum 114-2 *ΔerpΔPDE_03657*	*Δpku70*::*hph*; *Δerp*::*ptra*; *ΔPDE_03657*::*bar*	This study
P. oxalicum 114-2 *ΔpδΔPDE_00613*	*Δpku70*::*hph*; *Δpδ*::*ptra*; *ΔPDE_00613*::*bar*	This study
P. oxalicum 114-2 *ΔpδΔPDE_03657*	*Δpku70*::*hph*; *Δpδ*::*ptra*; *ΔPDE_03657*::*bar*	This study
P. oxalicum 114-2 *ΔPDE_00613ΔPDE_03657*	*Δpku70*::*hph*; *ΔPDE_03657*::*ptra*; *ΔPDE_00613*::*bar*	This study
BiFC-*erp*/*pδ*	*Pgpd*::*erp*::*eyfpN*::*TtrpC*::*hph*; *Pgpd*::*pδ*::*eyfpC*::*TtrpC*::*ptra*;	This study
BiFC-*erp*/*eyfpC*	*Pgpd*::*erp*::*eyfpN*::*TtrpC*::*hph*;	This study

	Pgpd::*eyfpC*::*TtrpC*::*ptra*;	
BiFC-*pδ*/*eyfpN*	*Pgpd*::*eyfpN*::*TtrpC*::*hph*;	This study
	Pgpd::*pδ*::*eyfpC*::*TtrpC*::*ptra*;	
BiFC-*eyfpN*/*eyfpC*	*Pgpd*::*eyfpN*::*TtrpC*::*hph*;	This study
	Pgpd::*eyfpC*::*TtrpC*::*ptra*;	

S1. Protein accession numbers in the phylogenetic tree in Fig. 1a.

Hp24γ2 accession number: NP_057124.2, Hp24γ1 accession number: NP_006849.1, Hp24γ4 accession number: NP_031390.1, Hp24γ3 accession number: NP_861974.1, Hp24γ5 accession number: NP_653277.1, Hp24β1 accession number: NP_006806.1, Hp24δ1 accession number: NP_006818.3, Hp24α2 accession number: NP_059980.2; Dcg31787 accession number: NP_724105.1, Dcg9053A accession number: NP_572994.1, Dcg1967 accession number: NP_572754.1, Dlogjam accession number: AAL28932.1, Dcg3564 accession number: NP_572165.1, Dcg9308 accession number: NP_611629.1, Declair accession number: NP_788616.1, Dbaiser accession number: NP_651323.3; Serp2 accession number: NP_009395.1, Serp4 accession number: NP_014659.1, Serp3 accession number: NP_010266.1, Semp24 accession number: NP_011315.1, Serv25 accession number: NP_013701.1, Serp1 accession number: NP_009402.1, Serp6 accession number: NP_011513.1, Serp5 accession number: NP_011978.1; Xp24γ3 accession number: NP_989261.1, Xp24γ5 accession number: AAH87777.1, Xp24γ1 accession number: NP_001039263.1, Xp24γ2 accession number: NP_001016560.1, Xp24α3 accession number: NP_001015779.1, Xp24δ2 accession number: NP_001007925.1; Pp24α1 accession number: XP_517060;

A3g07680.1 accession number: NP_187425.1, A3g29070.1 accession number: NP_189550.2, A2g03290.X accession numer: AAD17445.1, A1g69460.1 accession number: NP_177105.2, A1g21900.1 accession number: NP_173608.1, A1g09580.1 accession number: NP_172429.1. The tree was constructed by the neighbor-joining method. Bootstrap values are shown at each note, and are calculated from 1000 trees. Bar, evolutionary distance of 0.2.
